# Supplementary material for: Impact of Palliative Care in Evaluating and Relieving Symptoms in Patients with Advanced Cancer. Results from the DEMETRA Study
Source: Int J Environ Res Public Health. 2020 Nov 14;17(22):8429. doi: 10.3390/ijerph17228429 (PMC7698052; doi:10.3390/ijerph17228429)
Supplement: Supplementary file 1 [file ijerph-17-08429-s001.zip › ijerph-984170-suppl/suppl_table_1.pdf]

Supplementary Table S1. Number of symptoms simultaneously experienced by the 865 study patients at baseline.

Number of symptoms	Patients	
	N	%
≤4	68	7.9
5	57	6.6
6	75	8.7
7	87	10.1
8	89	10.3
9	109	12.6
10	65	7.5
11	64	7.4
12	85	9.8
13	39	4.5
14	42	4.9
≥15	85	9.8
