# Supplementary material for: Impact of Palliative Care in Evaluating and Relieving Symptoms in Patients with Advanced Cancer. Results from the DEMETRA Study
Source: Int J Environ Res Public Health. 2020 Nov 14;17(22):8429. doi: 10.3390/ijerph17228429 (PMC7698052; doi:10.3390/ijerph17228429)
Supplement: Supplementary file 1 [file ijerph-17-08429-s001.zip › ijerph-984170-suppl/suppl_table_2.pdf]

Supplementary Table S2. Most frequent combinations of concomitant symptoms among 865 patients at baseline.

Symptoms	N symptoms	Patients	
		N	%
Asthenia, Poor well-being	2	595	68.9
Asthenia, Lack of appetite	2	591	68.3
Asthenia, Drowsiness	2	525	60.7
Lack of appetite, Poor well-being	2	518	60.0
Lack of appetite, Asthenia, Poor well-being	3	507	58.6
Asthenia, Pain	2	499	57.7
Asthenia, Depression	2	466	53.9
Asthenia, Drowsiness, Poor well-being	3	451	52.1
Asthenia, Drowsiness, Lack of appetite	3	450	52.0
Pain, Poor well-being	2	442	51.1
Asthenia, Nausea	2	440	50.9
Pain, Poor well-being, Asthenia	3	430	49.7
Pain, Lack of appetite	2	425	49.1
Pain, Lack of appetite, Asthenia	3	416	48.1
Asthenia, Drowsiness, Poor well-being, Lack of appetite	4	400	46.2
Pain, Lack of appetite, Poor well-being, Asthenia	4	374	43.2
