# Supplementary material for: Impact of Palliative Care in Evaluating and Relieving Symptoms in Patients with Advanced Cancer. Results from the DEMETRA Study
Source: Int J Environ Res Public Health. 2020 Nov 14;17(22):8429. doi: 10.3390/ijerph17228429 (PMC7698052; doi:10.3390/ijerph17228429)
Supplement: Supplementary file 1 [file ijerph-17-08429-s001.zip › ijerph-984170-suppl/suppl_table_3.pdf]

Supplementary Table 3. Prevalence of symptoms experienced by 865 patients at baseline in relation to four major primary tumor sites.

**Symptoms**	**Primary tumor site, prevalence of symptoms (%)**			
	**Lung (N=151)**	**Pancreas (N=76)**	**Colon-rectum (N=75)**	**Breast (N=50)**
Asthenia	89.4	89.5	90.7	88.0
Poor well-being	72.8	75.0	73.3	66.0
Lack of appetite	71.5	75.0	77.3	66.0
Drowsiness	64.2	57.9	69.3	76.0
Pain	66.9	69.7	66.7	62.0
Depression	57.0	68.4	61.3	58.0
Anxiety	57.6	57.9	45.3	68.0
Breathlessness	59.6	35.5	40.0	30.0
Nausea	35.8	55.3	48.0	40.0
