# Supplementary material for: Impact of Palliative Care in Evaluating and Relieving Symptoms in Patients with Advanced Cancer. Results from the DEMETRA Study
Source: Int J Environ Res Public Health. 2020 Nov 14;17(22):8429. doi: 10.3390/ijerph17228429 (PMC7698052; doi:10.3390/ijerph17228429)
Supplement: Supplementary file 1 [file ijerph-17-08429-s001.zip › ijerph-984170-suppl/suppl_table_4.pdf]

Supplementary Table 4. Changes in intensity of main symptoms between day 0 and day 7 in 508 patients with severe degree of intensity at baseline.

Symptoms	N patients	Intensity, mean (SD)			p value[a]
		Day 0	Day 7	Difference (Day 7 – Day 0)	
Asthenia	282	6.74 (1.63)	5.50 (2.43)	-1.24 (2.41)	<0.001
Poor well-being	222	6.55 (1.41)	5.21 (2.38)	-1.34 (2.41)	<0.001
Lack of appetite	194	6.86 (1.66)	5.17 (2.91)	-1.69 (2.83)	<0.001
Drowsiness	150	6.51 (1.49)	4.57 (2.74)	-1.94 (2.75)	<0.001
Pain	162	6.16 (1.31)	3.96 (2.37)	-2.20 (2.57)	<0.001
Depression	136	6.63 (1.62)	5.04 (2.57)	-1.58 (2.55)	<0.001
Anxiety	109	6.47 (1.50)	4.86 (2.49)	-1.61 (2.39)	<0.001
Breathlessness	104	6.56 (1.59)	5.00 (2.75)	-1.56 (2.46)	<0.001
Nausea	71	6.46 (1.56)	4.03 (3.02)	-2.44 (3.02)	<0.001

NS, not statistically significant; SD, standard deviation. [a]p value for difference between day 7 and day 0.
