# Supplementary material for: Impact of Palliative Care in Evaluating and Relieving Symptoms in Patients with Advanced Cancer. Results from the DEMETRA Study
Source: Int J Environ Res Public Health. 2020 Nov 14;17(22):8429. doi: 10.3390/ijerph17228429 (PMC7698052; doi:10.3390/ijerph17228429)
Supplement: Supplementary file 1 [file ijerph-17-08429-s001.zip › ijerph-984170-suppl/suppl_table_5.pdf]

**Supplementary Table 5.** Changes in symptom prevalence between day 0 and day 7 in 508 patients according to care setting.

| Symptoms | Setting | Prevalence, N (%) | | |
|---|---|---|---|---|
| | | Day 0 | Day 7 | p value[a] |
| Asthenia | Home | 348 (93.5) | 352 (94.6) | NS |
| | Hospice | 85 (92.4) | 81 (88.0) | NS |
| | Hospital | 39 (88.6) | 41 (93.2) | NS |
| | p value[b] | NS | NS | |
| Poor well-being | Home | 302 (81.2) | 302 (81.2) | NS |
| | Hospice | 63 (68.5) | 66 (71.7) | NS |
| | Hospital | 31 (70.5) | 31 (70.5) | NS |
| | p value[b] | <0.001 | <0.01 | |
| Lack of appetite | Home | 280 (75.3) | 280 (75.3) | NS |
| | Hospice | 68 (73.9) | 56 (60.9) | <0.05 |
| | Hospital | 26 (59.1) | 26 (59.1) | NS |
| | p value[b] | NS | <0.01 | |
| Drowsiness | Home | 264 (71.0) | 263 (70.7) | NS |
| | Hospice | 64 (69.6) | 63 (68.5) | NS |
| | Hospital | 29 (65.9) | 25 (56.8) | NS |
| | p value[b] | NS | <0.05 | |
| Pain | Home | 267 (71.8) | 252 (67.7) | NS |
| | Hospice | 45 (48.9) | 42 (45.7) | NS |
| | Hospital | 31 (70.5) | 25 (56.8) | NS |
| | p value[b] | <0.001 | <0.001 | |
| Depression | Home | 224 (60.2) | 234 (62.9) | NS |
| | Hospice | 67 (72.8) | 63 (68.5) | NS |
| | Hospital | 13 (29.5) | 22 (50.0) | <0.05 |
| | p value[b] | <0.001 | NS | |
| Anxiety | Home | 209 (56.2) | 230 (61.8) | <0.05 |
| | Hospice | 56 (60.9) | 48 (52.2) | NS |
| | Hospital | 25 (56.8) | 23 (52.3) | NS |
| | p value[b] | NS | NS | |
| Breathlessness | Home | 182 (48.9) | 151 (40.6) | <0.001 |
| | Hospice | 36 (39.1) | 29 (31.5) | NS |
| | Hospital | 15 (34.1) | 12 (27.3) | NS |
| | p value[b] | NS | NS | |
| Nausea | Home | 187 (50.3) | 168 (45.2) | <0.05 |
| | Hospice | 30 (32.6) | 17 (18.5) | <0.01 |
| | Hospital | 8 (18.2) | 7 (15.9) | NS |
| | p value[b] | <0.001 | <0.001 | |

NS, not statistically significant; SD, standard deviation. [a]p value for difference between day 7 and day 0; [b]p value for difference between care settings.
